# Understanding the delayed prescribing of antibiotics for respiratory tract infection in primary care: a qualitative analysis

**DOI:** 10.1136/bmjopen-2016-011882

**Published:** 2016-11-18

**Authors:** R Ryves, C Eyles, M Moore, L McDermott, P Little, G M Leydon

**Affiliations:** 1 Department of Primary Care & Population Sciences, University of Southampton, Southampton, UK; 2 Department of Primary Care & Population Health Sciences, King's College London, London, UK

**Keywords:** PRIMARY CARE, Antibiotics, Respiratory Tract Infection, Prescribing, QUALITATIVE RESEARCH

## Abstract

**Objective:**

To identify general practitioner (GP) views and understanding on the use of delayed prescribing in primary care.

**Design:**

Qualitative semistructured telephone interview study.

**Setting:**

Primary care general practices in England.

**Participants:**

32 GPs from identified high-prescribing and low-prescribing general practices in England.

**Method:**

Semistructured telephone interviews were conducted with GPs identified from practices within clinical commissioning groups with the highest and lowest prescribing rates in England. A thematic analysis of the data was conducted to generate themes.

**Results:**

All GPs had a good understanding of respiratory tract infection (RTI) management and how the delayed prescribing approach could be used in primary care. However, GPs highlighted factors that were influential as to whether delayed prescribing was successfully carried out during the consultation. These included the increase in evidence of antimicrobial resistance, and GPs' prior experiences of using delayed prescribing during the consultation. The patient–practitioner relationship could also influence treatment outcomes for RTI, and a lack of an agreed prescribing strategy within and between practices was considered to be of significance to GPs. Participants expressed that a lack of feedback on prescribing data at an individual and practice level made it difficult to know if delayed prescribing strategies were successful in reducing unnecessary consumption. GPs agreed that coherent and uniform training and guidelines would be of some benefit to ensure consistent prescribing throughout the UK.

**Conclusions:**

Delayed prescribing is encouraged in primary care, but is not always implemented successfully. Greater uniformity within and between practices in the UK is needed to operationalise delayed prescribing, as well as providing feedback on the uptake of antibiotics. Finally, GPs may need further guidance on how to answer the concerns of patients without interpreting these questions as a demand for antibiotics, as well as educating the patient about antimicrobial resistance and supporting a good patient–practitioner relationship.

**Strengths and limitations of this study**

- Using a purposive sample of high-prescribing and low-prescribing practices in England captured a broad view of delayed prescribing strategies in primary care.
- Individual prescribing data were not available and this may have been beneficial to ensure maximum variance of the sample interviewed.
- Recruitment to the study was difficult, but the sample collected was varied according to key parameters and the findings resonate with other published research, which increases confidence in face validity and transferability of the findings.

## Background

Respiratory tract infections (RTIs) are usually brief and self-limiting conditions, with antibiotics having little or no clinical benefit, unless there is a serious underlying comorbidity.^1^ However, UK figures have revealed that recent falls in prescribing have stalled,^2^ with RTI accounting for ∼60% of all prescriptions in primary care.^3^ ^4^ Prescribing unnecessary antibiotics can be a drain on the National Health Service (NHS) resources, and can have serious consequences to patient health, including the risk of side effects and the antimicrobial resistance (AMR).^5^ ^6^ AMR has been described by the WHO as a serious issue that must be addressed with some urgency,^7^ as well as being cited as a ‘catastrophic threat’ by the UK's Chief Medical Officer.^8^

The delayed prescribing of antibiotics for RTI is a technique that can be used by health professionals to reduce unnecessary prescribing in primary care.^9^ Delayed prescribing is a method whereby a prescription is issued by a health professional for use by the patient at a later date, if their symptoms do not improve. The National Institute for Health and Care Excellence (NICE) guidelines have recommended this strategy as a management option for most patients presenting with RTI, unless they meet a specific criteria of risk factors that signal potential complications.

The use of delayed prescribing can limit the collection of prescriptions by patients to just 40%.^9–11^ Research shows that delayed antibiotic prescriptions are as effective as immediate prescriptions in reducing complications from RTI, as well as reducing the need for patient reconsultation;^12^ which suggests that delay offers a reasonable alternative to an immediate prescription. In addition, delayed prescribing has the potential to be more effective in reducing antibiotic use for RTI, so long as health professionals provide clear treatment advice to patients.^9^ ^13^ A Cochrane review of the use of delayed antibiotic prescribing compared with immediate or no prescribing found there were no differences between the strategies for clinical outcome; with delayed prescribing resulting in a significant reduction in antibiotic use compared with immediate prescribing.^11^ However, there is some evidence to suggest that patient satisfaction concerning the outcome of consultations appears to be lower in those receiving a delayed prescription compared with those being issued one immediately.^9^

Reports on antibiotic prescribing suggest that the delayed prescribing technique may not be widely or consistently used by general practitioners (GPs).^2^ Data have illustrated large differences in antibiotic use across the country; however, these rates have only been observed and not explained.^2^ ^4^ Moreover, some GPs have been reported to be critical of the delayed prescribing approach when faced with uncertainty about prognosis and handing over decision-making to patients.^14^ Perceived patient expectations for antibiotics may also influence GP prescribing behaviours.^15^ ^16^ Given these complex contextual factors, delayed prescribing can potentially be an effective prescribing strategy in providing reassurance for the GP and the patient, by making antibiotics available should symptoms worsen, and in terms of validating patient concerns.^17^

The aim of this study was to identify GP views on the use of delayed prescribing, their use of the technique and factors that can enhance or inhibit its use in routine general practice. In addition, the study aimed to elicit GPs' views on current prescribing guidelines, and what information would be beneficial if training were to be provided. It was anticipated that the information gathered from the study could be used to inform the future development of an intervention to aid effective delayed antibiotic prescribing.

## Methods

A qualitative research design using semistructured telephone interviews was conducted with GPs from high-prescribing and low-prescribing primary care practices in England. Research Ethics Committee (REC) approval was obtained from the host research site (Hampshire and Isle of Wight) in November 2013.

Practices were identified using National Prescribing Data available through the NHS Business Services Authority Portal.^18^ Eight clinical commissioning groups (CCGs) with the highest and lowest prescribing data were selected according to specific therapeutic group age–sex related prescribing units data.^19^ The 10 practices with the highest prescribing data within each of the high-prescribing CCGs, and the 10 practices with the lowest data within each of the low-prescribing CCGs were selected. Walk-in centres were excluded from the study as the research was aimed to understand prescribing behaviours in general practice. It was anticipated that this primary sampling approach of identifying high-prescribing and low-prescribing practices would facilitate maximum variation sampling to get a range of differing GP views about the use of antibiotics for RTI and delayed approaches to prescribing. A target of 30–50 GPs was estimated (15–25 GPs from each group), or until saturation was indicated through data analysis.

Practices were telephoned by RR to elicit interest in taking part in the study. Interested GPs were sent an electronic participant information leaflet (PIL) with a reply slip and consent form. Initial recruitment to the study was slow; therefore, an invitation letter from the Royal College of General Practitioners (RCGP) National Clinical Champion for Antimicrobial Stewardship (co-author MM) was sent together with the PIL and consent form to all GPs within identified practices, using the Docmail mailing service. Clinical research networks also contacted practices on behalf of the research team to help with recruitment. Once written informed consent was received RR made contact with GPs to schedule a time for a telephone interview. GPs were offered £50 as payment for taking part.

A semistructured interview topic guide was developed (see online supplementary 1) building on questions from a topic guide for a study exploring GP views in relation to antibiotic prescribing in infection.^17^ The guide aimed to elicit GP views and experiences of using delayed prescribing in practice for RTI, as well as perceived barriers and facilitators of implementing this technique. GPs were invited to discuss their opinions of having training in using delayed antibiotic prescribing strategies. The guide was piloted with a practising GP to ensure the acceptability of the questions asked and the feasibility of completing interviews within a 30 min timeframe. The topic guide changed as new ideas emerged from early data collection, but the general focus of the interviews remained the same. RR conducted all interviews. Interviews were recorded, anonymised and transcribed verbatim.

10.1136/bmjopen-2016-011882.supp1 Supplementary file

**Figure 1:**
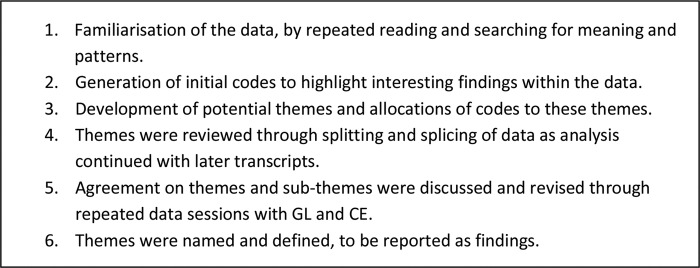
Stages of data analysis.

Interview data were analysed using thematic analysis.^20^ An inductive approach was adopted because this was a relatively unexplored area that required researchers to be open to new insights. RR led the analysis and developed the initial codes. The reviewing and agreement of themes were discussed and confirmed through repeated data sessions with GML and CE, who are both experts in qualitative research. The stages of the analytic process are shown in figure 1. Thematic saturation was achieved when no new themes were emerging from new data.

## Findings

### Participants

**Figure 2:**
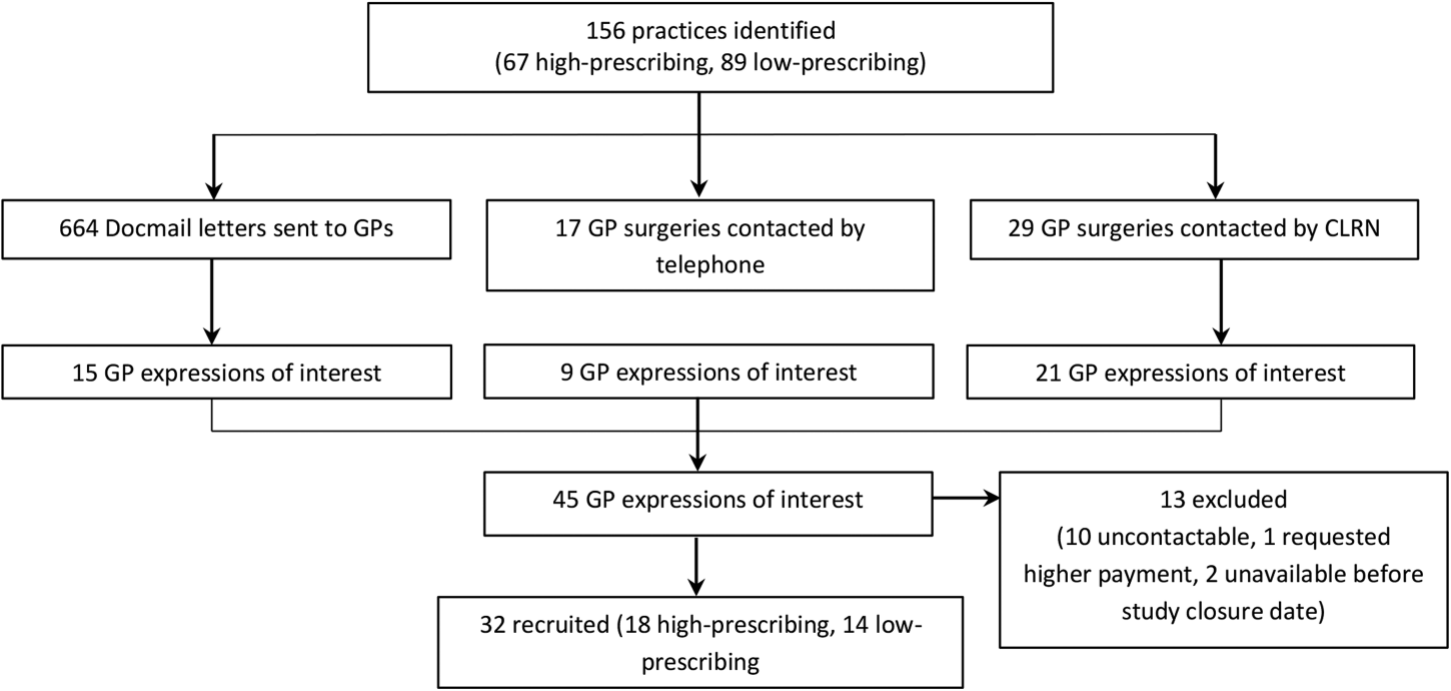
Recruitment flow chart. CLRN, Comprehensive Local Research Network; GP, general practitioner.

Figure 2 illustrates the process of recruiting GPs to the study.

Table 1 provides demographics of the CCGs and practices from which GPs were recruited. The prescribing level was relevant to the analysis as accounts from GPs of high-prescribing and low-prescribing practices were compared to identify any similarities or differences between the groups. Interviews with the 32 GPs lasted between 12 and 34 min (M=25.08).

**Table 1:** CCG and practice demographics

CCG	Prescribing level	Practices recruited	Average practice list size	Average practice deprivation score*	GP total
Brighton and Hove	Low	2	8992	4	2
County Durham and Tees Valley	High	9	9104	5	7
Derbyshire	High	3	10 220	8	1
London	Low	6	9896	6	3
Newcastle West	Low	6	9097	4	5
South of Tyne and Wear	High	1	9963	2	1
Trent	Low	2	9212	3	2

Data sourced from DCLG.

*Index of Multiple Deprivation Scores, where lower scores=higher deprivation.

CCG, clinical commissioning group; DCLG, Department of Communities and Local Government; GP, general practitioner.

### Themes

Four major themes and nine subthemes were identified (table 2). The themes and subthemes are discussed with illustrative quotations. The GP identification number and whether they are from a high-prescribing or low-prescribing practice are included at the end of each quotation.

**Table 2:** Themes and subthemes derived from thematic analysis of the data

Theme	Subtheme
1. Management of RTI in a GP consultation	1.1 Understanding RTI 1.2 Patient assessment during consultation
2. Understanding and uses of delayed antibiotic prescribing	2.1 Delayed antibiotic prescribing in the consultation 2.2 GP attitude towards delayed prescribing
3. Factors that influence the use of delayed antibiotic prescribing	3.1 Influences on GP decision-making 3.2 Protecting the patient–practitioner relationship 3.3 Guidelines and policies
4. Views on the need for training	4.1 What training should be given 4.2 Training delivery

GP, general practitioner; RTI, respiratory tract infection.

#### Management of RTI in a GP consultation

GPs were invited to discuss their understanding of RTI and how symptoms could be self-managed by patients. In addition, GPs described their views on the use and efficacy of prescribing for RTI.

##### Understanding of RTI

All GPs understood that most upper RTIs were viral and self-limiting, with most symptoms clearing without the need for antibiotics.

The vast majority are viral and it's best to avoid antibiotics, except in extreme cases. Most antibiotics don't benefit most conditions. (GP5, high prescribing)

The majority of GPs would try to discuss the natural history of RTI with their patients in order to help build the evidential basis for no antibiotic disposal. All GPs described suitable alternative treatments for the self-management of RTI symptoms, and described routinely trying to inform their patients of these.

Three things that I use is if they drink a lot of water, take a lot of rest and take paracetamol on and off with a fever, most of the time it's a supporting measure as the body fights very well. (GP12, high prescribing)

However, a few GPs explained that they did not offer alternative advice or treatment strategies to their patients due to the pressure of time constraints:

I don't have time really. Most GP consultations are ten minutes long and there's not actually that much time to take a history, examine a patient properly, to go through the findings, what the plan will be and then to then go through the ways of managing. (GP22, high prescribing)

##### Patient assessment during consultation

Treatment decisions were primarily determined through examination and history of the patient. If a patient had a serious underlying condition, GPs would be more likely to prescribe antibiotics if they felt the patient was at higher risk of developing an infection. As well as comorbidities, some GPs explained that the age of patients would guide their decision:

There are other patients who are more vulnerable like the elderly, people with concomitant conditions as well that might make them more vulnerable. So those are the group of patients that I think NICE was talking about excluding in terms of either prescribing antibiotics or this concept of delayed antibiotics. (GP24, high prescribing)

GPs described a heightened sense of obligation to issue antibiotics to children compared with adults due to greater levels of uncertainty about the severity of their symptoms. Others reported that they felt a greater pressure to prescribe antibiotics when confronted with a concerned parent:

With parents the impression you get is ‘You need to give something to make my child better’. If you're not giving them something to make them better then you're making them worse or you're not helping.(GP22, low prescribing)

Some GPs acknowledged that the risk of issuing antibiotics for an RTI outweighed the potential benefits, and they would explain this to their patients in the consultation to try to avoid unnecessary prescribing.

Then going through the discussion that there's lots of evidence nowadays to say that antibiotic prescriptions don't reduce complications, they barely reduce the duration of the illness, and if it is it's only by a few hours. Then you've got to weigh that up against the possible side effects of taking the medication. (GP20, high prescribing)

#### Understanding and uses of delayed antibiotic prescribing

All GPs conveyed understanding of delayed antibiotic prescribing, different methods of issue and its appropriate use for patients presenting with an RTI. Two subthemes encompass the views expressed: the use of delayed antibiotic prescribing in the consultation and GP attitudes towards delayed antibiotic prescribing.

##### Delayed antibiotic prescribing in the consultation

GPs were invited to provide their own definition and method of delayed prescribing. All GPs perceived delayed antibiotic prescribing as a strategy that could be used when they believed that antibiotics might not be needed by the patient at the time, but a prescription should be issued as a precaution:

This is trying to explain to patients that this is what is going on, and this is the progression that we would expect for the illness, and that there's no indication for giving antibiotics at this stage, but if x, y, and z happens then it may be worth cashing the prescription and starting the antibiotics. (GP9, high prescribing).

All GPs were able to identify at least one method of how to delay an antibiotic prescription (table 3), and all GPs reported that they had used delayed prescribing on a regular basis when needed.

**Table 3:** GPs' preferred delayed prescribing strategy

Delayed antibiotic prescribing strategy	High prescribing	Low prescribing	Total
Issue prescription in consultation and advise when to collect	10	13	23
Issue postdated prescription in consultation	3	1	4
Offer recontact with clinician to agree release of prescription if symptoms worsen	3	0	3
Collect prescription from reception if symptoms worsen	0	1	1
Issue prescription for patients to take antibiotics then consult over telephone to stop or continue course	1	0	1

GP, general practitioner.

The majority of GPs would issue a prescription during the consultation, and advise patients to only cash it in if their symptoms were to worsen. Commonly short delays were used—shorter than the likely natural history of the infections, or than strategies used in previous trials:

Delayed antibiotic prescribing, from my point of view, is where I provide the prescription for an antibiotic with a date at some point in the future, normally 24 or 48 hours thereafter. With instructions to the patient, or the family, as to the indicators for cashing the prescription and using it. (GP10, high prescribing)

As well as postdating prescriptions, another strategy described was leaving the prescription at reception for patients to collect:

I put a nice big D on the right hand side and just drop it in the box. The staff then know that that's a delayed prescription and I can say to a patient ‘I'll leave it out for you at reception’, […] if it's not been prescribed within a couple of days, an appropriate length of time, then it will be taken out. (GP17, low prescribing)

Alternatively, a few GPs would not provide a prescription during the consultation, but would advise reconsultation if symptoms did not self-limit:

For me, it would be not to prescribe in the morning of the consultation, and to prescribe three or four days later because there have been some changes in the symptoms of the patient that shows me that there is some evidence now that it could be beneficial to use some antibiotics. (GP11, high prescribing)

GPs from high-prescribing and low-prescribing practices expressed uncertainty as to whether they were adopting the correct approach, which technique was the most effective, or were ‘not quite sure’ (GP7, high prescribing) of other methods. One GP suggested that delayed prescribing strategies were ‘just too complicated’ (GP22, low prescribing).

When asked about their prescribing rate, GPs were uncertain about how many delayed antibiotic prescriptions were cashed in. Some GPs felt that it would be a useful figure to know to see whether their delayed prescribing method was effective, which in turn would justify their use of the strategy:

I have no idea how many of these delayed prescriptions are cashed in, which is a great shame because it would be very nice […] it would be very interesting, wouldn't it? (GP1, high prescribing)

##### GP attitude towards delayed prescribing

Despite some reservations (as above), GPs generally had a positive attitude towards delayed prescribing. They described it as a practical, convenient tool for the GP and the patient and that it was primarily used to ‘avoid unnecessary antibiotic prescription’ (GP12, high prescribing). It was hoped that by using a delayed approach, patients would not cash in the prescription.

GPs explained that issuing a delayed prescription was practical for patients as it would enable them to access antibiotics if their symptoms were to worsen or not improve, without the need for reconsultation. This may be down to issues of travel, or time constraints from the patient and practice. Some GPs reported a greater perceived demand to prescribe during consultations at the end of the week, thus delayed prescribing was a useful tool to relieve this pressure:

[On a] Friday afternoon it's a useful thing to give because of the difficulty sometimes in accessing medical out-of-hours services, which can be a bit complex and geographically can be quite awkward if people who used to be seen locally now have to travel 15 miles to be seen. (GP26, low prescribing)

One GP explained that delayed prescribing offered a compromise when the GP was of the opinion that antibiotics were not appropriate but the patient desired an antibiotic prescription. If GPs were encountering difficulties in ending a consultation then a delayed prescription was described as a closing strategy.

I think it's often used in a situation where the clinician doesn't think antibiotics are justified, but the patient is adamant that they would like them and it's seen as a compromise. So it's basically a way to end the consultation where both parties can feel happy that they've got what they wanted. (GP31, high prescribing)

Some GPs described how delayed prescribing as a compromise elicited positive outcomes for the GP and the patient. One GP explained that patients felt they had been listened to. It also provided the GP with a safety net, in case they had missed signs of a more serious infection, as well as alleviating the fear of making the wrong diagnosis:

…they like to have a bit of paper when they leave us. I think there's a little bit in my mind as well…like I say where I've had one or two where people have usually three or four weeks down the line actually deteriorated but still blame me for the fact that I didn't give something at that time. (GP25, low prescribing)

However, some GPs found particular methods of delayed prescribing to be ‘mutually inconvenient’ (GP13, low prescriber) for the GP and the patient. One GP thought that if the method of telephone reconsultations was used, this created more work for the patient and the GP. Delayed prescribing could also potentially be confusing for the patient, as the instructions risked being viewed as vague or unclear. As a result, one GP suggested that delaying a prescription might neither be safe nor sensible:

I think sometimes it actually muddies the water and that's why I've sometimes thought it's actually better not to do it […] unless you give them a really specific guideline it can be quite vague. (GP31, low prescribing)

Moreover, a couple of GPs thought that delayed prescribing conveyed a contradictory message to patients:

I feel that to be saying, ‘There's no indication for antibiotics at this stage, but if you're no better in 48 hours then by all means take them’ is a mixed message for me. (GP9, high prescribing)

#### Factors that influence the use of delayed antibiotic prescribing

GPs were invited to reflect on factors that acted as facilitators or barriers to using delayed antibiotic prescribing. Factors included influences on GP decision-making, protecting the patient–practitioner relationship, and the use of guidelines and policies.

##### Influences on GP decision-making

One factor that influenced GPs' treatment decisions for RTI was a culmination of the increase in evidence of AMR and the experience they had gained in practice. In particular, GPs from practices with low-prescribing rates felt that this had helped reduce their prescribing:

Experience, training in the past and also over the last 15 years there's been a lot of discussion and debate about antibiotics in the medical journals on courses, and I think over the years, […] certainly my antibiotic prescribing has changed and we have antibiotic prescribing audits within the practice. Our prescribing is reducing. (GP23, low prescribing)

Those GPs with higher levels of experience expressed higher perceptions of self-efficacy in using delayed prescribing strategies:

As time goes by I think you develop better explanations for patients that mean when you are explaining to them why they don't need an antibiotic you are more convincing. (GP31, low prescribing)

However, some GPs from high-prescribing practices found delayed prescribing strategies were complex and the uncertainty in administering them led to difficulties in successfully delaying a prescription. These difficulties included being able to fully understand the method of delayed prescribing and the confidence in using the strategy during the consultation, particularly when met with perceived patient pressure for an antibiotic prescription. In contrast, GPs from low-prescribing practices explained that using a delayed antibiotic prescription was useful as a precautionary measure and using some caution when treating a patient helped manage this uncertainty:

If you've clinically got somebody with you who you do think's unwell, but you're still not really getting clear symptoms of a bacterial infection, then yes, to manage your uncertainty. (GP21, low prescribing)

Another influential GP factor was the variation in prescribing rates and management of RTI among GPs within and between practices in the country. Consequently, GPs explained patients may be provided with conflicting treatment strategies from different GPs. GPs described the negative impact this could have on them educating their patients about the natural history of RTI and when antibiotics would be appropriate. GPs from high-prescribing and low-prescribing practices felt that it was important to spend time discussing antibiotic prescribing issues within the professional community to ensure that patients receive consistent treatment and advice:

When patients do return to come and see you often it's because they aren't clear on what's going on, or they've got mixed messages from different doctors, from that point of view, and I think certainly to present a common ground, sort of like a united front, in terms of policy, I think can be useful. (GP10, high prescribing)

Another challenge GPs faced was by their patients accessing out-of-hours care, where GPs felt that antibiotics for RTI were more readily available. The doctors at the walk-in centres would have no history of the patient and would therefore be more likely to give out antibiotics rather than educating the patient.

They [patients] often go to the out-of-hours centre and the out-of-hours centre does tend to give out antibiotics and I think that's entirely understandable because they don't know these people. (GP 1, high prescribing).

Overall, GPs described prescribing as a strategy to negotiate patient pressure due to social arrangements, comorbidities and age, as well as reducing the need to reconsult with patients.

##### Protecting the patient–practitioner relationship

GP accounts suggested that the relationship with their patients was a major influence on whether a delayed antibiotic prescription would be issued during a consultation. The majority of GPs described how perceived patient demand played a large role during the consultation and felt pressured into issuing a prescription if patients attended the consultation with an expectation for an antibiotic. Interviewees defined patients with a previous (successful) experience with an antibiotic for RTI as most likely to expect an immediate prescription for a new episode.

When you see them for the next time they have sinusitis or sinus pain, expect to be given antibiotics and then your consultation goes badly from the word go because that patient's expecting antibiotics and suddenly they're coming up against a barrier and somebody who's suggesting we don't use antibiotics. (GP21, low prescribing)

GPs would appease or mitigate this patient demand by spending time during the consultation explaining the natural history of RTI, ineffectiveness of antibiotics in treating the majority of upper RTIs and that the risks of taking antibiotics outweighed the benefits. Most GPs felt that spending time educating and reassuring patients that RTI symptoms should clear over time, and to use alternative treatments (such as analgesics and rest) facilitated their consultation.

A couple of GPs also expressed concern over receiving complaints from patients for not prescribing antibiotics. This in turn would make GPs more likely to prescribe. These concerns were preponderantly expressed by GPs from practices with high-prescribing rates:

I've had a complaint because I didn't prescribe an antibiotic once and that changes the way you prescribe because you don't want to have a complaint against you even if you are right. (GP8, high prescribing)

Overall, GPs described the use of delayed prescribing as a useful tool to help maintain a strong patient–practitioner relationship.

If the explanation that's gone with the delayed prescription is adequate and reasonable and there's been a decent consultation where everyone's felt involved and understands the reasons why the outcome is the outcome, generally it's a positive thing. (GP 10, high prescribing)

Some GPs explained that the initial consultation would allow for a form of shared decision-making, through negotiation and educating the patient about RTI in order to come up with an appropriate treatment plan. GPs described how spending time in the consultation to devise a treatment strategy and in some cases issue a delayed antibiotic prescription provided the patient with sufficient leverage to make an informed, (semi)-autonomous decision about the next steps in their treatment for RTI.

…sometimes they're much more receptive to the delayed prescription of it, and giving them back the autonomy: if they are the more self-caring, if you like, set of patients, who have given it a significant amount of time. (GP10, high prescribing)

Strategies GPs used to ensure patients were aligned with their treatment disposal included offering paper-based or web-based information leaflets, as well as providing reassurance to the patient by doing a clinical examination and clearly explaining their findings.

However, GPs emphasised that giving patient complete autonomy on the management of their RTI was reliant on how ‘trustworthy’ the patient was, which in turn would influence their decision as to whether to issue a delayed prescription. A minority of GPs described delayed prescribing as a poor strategy because it gave too much power to the patient by ‘leaving decisions in the patient's own hands’ (GP5, high prescribing). These GPs conveyed a paternalistic practice style as appropriate when treating RTI:

That's why you're a doctor and what you're paid for, so it's better for you to make the decision. (GP5, high prescribing)

##### Guidelines and policies

The majority of GPs were aware of guidelines (eg, NICE guidelines, Centor Criteria) that had been created to assist them with the management of RTI and advise the use of delayed antibiotic prescribing where appropriate. However, no GPs mentioned any current initiatives that were in place to encourage antimicrobial stewardship (such as the RCGP TARGET Toolkit^21^).

Some GPs suggested that there were so many different guidelines and policies; the amount of information was almost overwhelming:

We're bombarded with so many different guidelines for so many different things. Trying to keep up with all that is really tricky. (GP13, low prescribing)

GPs tended to rely on guidance and policies provided by their local CCG. However, GPs did not always find these guidelines useful and hard to implement in practice, because of perceived patient expectation and demand for antibiotics. Moreover, some GPs highlighted that local policies were inconsistent between CCGs over the country and stressed the importance of having some simple, standardised guidelines that could be easily implemented to ensure consistent prescribing at a national level. A few GPs highlighted that this issue as prescribing differences between and within practices equated to a national variation of prescribing rates. One GP from a low-prescribing practice suggested that CCGs should be responsible for auditing prescribing rates and that reducing antibiotic prescribing become a national priority:

I think the CCGs need to have an assessment in their area and see what the volume of prescribing is, where it's coming from and I think it does need to be right across the board including the out of hours teams, the respiratory nurse specialists, the urgent care nurses. (GP16, low prescribing)

The findings suggest that GPs with greater belief in their experience and knowledge of how to implement delayed prescribing strategies were more confident in delaying an antibiotic prescription. Protecting the patient–practitioner relationship by educating the patient as well as discussing treatment options was beneficial. Finally, the consensus among GPs was that the guidelines and policies were inconsistent and sometimes overwhelming, with differences between and within practices and CCGs.

#### Views on the need for training

Participants were invited to share their opinions of whether training in how best to use delayed antibiotic prescribing would be useful for them, and if so, what training should be provided and how.

##### GP attitude towards training

There were mixed opinions with regard to tools or training to be provided to GPs to assist them with delayed prescribing in practice. Some were open to the idea of training with the caveat that it would have to be relatively easy and not time intensive. Some expressed reservations about the perceived utility of such training for all GPs.

It's difficult. I mean, I haven't had any training for delayed prescribing of antibiotics, it's just a kind of common sense scenario. If I had a proper training, yes, it would be helpful for me. Some GPs might feel it's totally inappropriate. (GP19, high prescribing)

Most GPs felt that training in a conventional classroom-based sense was not necessary, but described the potential benefit of policy and research updates or standardised policies:

Would I go if there was a training session on delayed prescribing? I have to say probably not. I'd rather have the written information, the protocols in place. (GP16, low prescribing)

##### What training should be given

There was no consensus as to what training should be given; however, GPs felt more standardised, simpler written information should be provided to give more structured guidance for antibiotic prescribing. In addition, a high proportion of GPs discussed the utility of having prescribing rates specific to their practice to clarify whether the practice and their own personal practice needed to be changed in any way. GPs demonstrated willingness to share such individual prescribing information with their colleagues.

GPs also suggested that training in antibiotic prescribing should be given to GP registrars:

I think training needs to be given to GPs, and particularly GP registrars and trainees, about where to prescribe antibiotics, because particular GP registrars learn their prescribing habits from the practices that they're in, and I think there is a tendency not to question what goes on. So if you go, as a GP trainee, to a practice where there's higher antibiotic prescribing you'll learn habits from them, rather than any sort of scientific approach. (GP5, high prescribing)

Some GPs suggested that training in communication was essential in order to improve their prescribing rates. Learning how to have an effective consultation with patients would facilitate the delayed antibiotic prescribing process:

[It is] about communication skills, working out what the patients actually want, and what their ideas and concerns and expectations are. (GP3, high prescribing)

##### Training delivery

GPs that were supportive towards training were uncertain as to how best to deliver it. Time pressure appeared to be the biggest concern but most GPs felt they would get best value out of having a face-to-face meeting with other health professionals within their CCG.

I think the best choice would be at CCG time-out meetings, or if somebody could come and give a talk, so there were loads of GPs there rather than going to individual practices. If there is a possibility of some hand-outs or leaflets about it, just for us to go through and find out, or maybe some information from the practice pharmacists who can then pass it on to the GPs. (GP18, high prescribing)

GPs described a need for such information and guidance to be provided by a credible source, such as microbiologists, or fellow GPs who have experience of treating patients on a day-to-day basis.

Although there were mixed opinions as to whether training would be beneficial, GPs felt that having some guidance of implementing these strategies into practice may be useful. Training should have a wider focus on communication with the patient. The delivery of training would need to be time-efficient, and delivered by a credible source.

## Discussion

This study explored GPs' views and understanding of delayed antibiotic prescribing in primary care. The findings illustrate that all GPs have a good understanding of RTI, and how to assess and treat the illness. All GPs were aware of and were generally positive about delayed prescribing, but felt that certain factors would sometimes make it hard to implement this strategy.

### Strengths and limitations

The findings offer a novel insight into GPs' prescribing practice for RTI in a primary care setting. However, one notable limitation was that individual prescribing behaviours of GPs were unknown. Therefore, the prescribing rates of the practices obtained from the National Prescribing Data may not reflect the prescribing rates of the GPs interviewed for the study. Having data illustrating individual prescribing rates of GPs would have been beneficial to recruit a more representative sample. Practices were approached and recruited according to their global prescribing rates (not isolated to RTI) so it may be possible that GPs had ‘good’ prescribing habits relating to RTI, but not to other infections, which could explain the practices' high-prescribing rate.

The recruitment of GPs to the study was low, despite a three-pronged approach to elicit expressions of interest (see figure 2). Low recruitment may have influenced the findings as respondents with greater familiarity or a more positive attitude towards delayed prescribing may have been more willing to take part in the study. However, more practitioners from high-prescribing practices were recruited, which may minimise this risk. Moreover, analytic saturation was reached and the findings reflect those of another qualitative study investigating delayed prescribing strategies in primary care.^14^ This increases the trustworthiness and transferability of the findings.

### Main findings

The findings suggest that despite GPs knowing the natural history of RTI, they may use this knowledge to provide a more nuanced approach to delayed antibiotic prescribing. Antibiotic prescriptions would be postdated with a considerably short delay compared with research suggesting delays of up to 14 days for some RTIs.^22^ While all GPs were able to provide a definition and at least one example of delayed antibiotic prescribing, the majority were unaware of alternative delaying methods. However, evidence suggests that the choice of method is not critical.^9^ GPs would find benefit in knowing how many delayed prescriptions were cashed in compared with immediate use prescriptions at an individual and practice level, to see whether particular delayed prescribing strategies are effective.

Despite generally positive attitudes towards delayed prescribing, there was a preference for no prescribing at all, which is the preferred strategy for management of infection.^11^ Delayed prescribing was seen as a compromise or a negotiation, either to meet a presumed patient expectation for antibiotics or to provide reassurance to the patient. GPs with greater experience or knowledge in using delayed antibiotic prescribing found the approach easier to use and manage uncertainty. Some GPs felt that the delayed approach maintained the patient–practitioner relationship by providing the patient with some form of autonomy in the management of their illness.

The findings further emphasise that patient expectation and clinician perception for antibiotics when presenting with an RTI could be influential, which corroborates with other research.^23^ Where an antibiotic prescription is not indicated, doctors will still issue a prescription during the consultation ‘just in case’ a patient's symptoms worsen.^24^ However, effective communication with and education of the patient about the natural history of RTI may be more beneficial in reducing the number of unnecessary antibiotic prescriptions. There is some empirical evidence to support the role of improved communication skills in reducing prescribing.^23^ ^25^ This could provide reassurance for the patient, as well as validating their illness, which in turn will enhance opportunities for maintaining a strong patient–practitioner relationship for future consultations.^15^ ^17^

The favoured delayed prescribing strategy was issuing a prescription during the consultation, advising patients to cash it in if symptoms worsen, which may be due to perceived patient demand and expectation for antibiotics.^26^ Despite a mixed response from GPs with regard to training in how to use delayed prescribing strategies effectively, there was some consensus of the need of a standardised policy between and within practices and CCGs to ensure that strategies are consistent throughout the country. GPs were uncertain as to how training should be delivered and it is likely that it would need to be flexible to suit local needs, using a variety of teaching modalities. One interesting finding was that GPs were unaware of existing tools that have been developed to assist with antibiotic prescribing in primary care, such as the RCGP TARGET Antibiotics Toolkit,^21^ an online resource to encourage responsible antibiotic prescribing in primary care.

There appears to be a lack of transparency regarding antibiotic prescribing within and between primary care practices in England. Clear, shared goals are required and can be aided by some form of training informed by national and local guidance. Prescribing practice is the result of interaction between two parties: the patient and the GP, with effective communication at the heart of the prescribing relationship. Negotiation is an important tool for achieving a successful outcome, but there is insufficient knowledge as to how best GPs can achieve an optimal negotiation with individual patients and for the wider population. The lack of feedback of the results of delayed prescribing were highlighted as one problem eroding motivation, this could be addressed through improved information technology.

### Comparison with existing literature

Few qualitative studies have explored the use of delayed prescribing in UK primary care; however, the main findings of this study resonate with some of the findings of one study exploring the usefulness of the delayed prescribing strategy.^14^ Both studies highlight that perceived patient expectation makes consultations more challenging, as well as the use of delayed prescribing to manage uncertainty during a consultation, as a safety net, and to maintain the patient–practitioner relationship. Our findings further suggest that GPs with a greater understanding of delayed prescribing strategies and experience of using it are more positive towards the approach. Moreover, the current study suggested that GPs may not be taking into account the variation in the natural history of RTI and would issue a delayed prescription with a short delay regardless of the anticipated duration of the illness. Lower RTI lasts on average 12 days following the index consultation and there is unlikely to be a substantial improvement with 24–48 hours, which in turn may result in collection of unnecessary antibiotics.^22^

Qualitative research conducted in other countries has demonstrated similar findings to the current study.^27–30^ Evidence suggests that GP prescribing decisions depend on the interaction with the patient and other influencing factors, such as GP characteristics and continuity of care, mutual trust, and flexibility with the patient.^30^ GPs interviewed for the current study also saw prescribing as a negotiation tool; however, some did not agree with the delayed prescribing approach as they found it to be contradictory and felt patients ought to have less autonomy.

In addition, the setting of the encounter was of importance to GPs.^30^ GPs found that having consistencies in prescribing behaviours throughout the primary care centre, as well as the use of local professional discussions and exchange of experience that facilitates their prescribing practice. This emphasises the call for wider policy implementation and open discussion within and between practices within CCGs, to enable a more consistent rate of prescribing around the UK.

However, we confirmed the concerns of some prescribers who perceive delayed prescribing to be an unhelpful management strategy for patients with self-limiting RTIs.^14^ Suitable training could potentially address these concerns to enable prescribers to use delayed prescription appropriately and manage consultations more effectively. Other research has suggested that to reduce antibiotic prescribing, interventions need to include several factors such as education about appropriate RTI management, making strategies more acceptable to GPs, which in turn may improve the effectiveness of their implementation.^31^ ^32^

### Implications for policy, research and practice

The findings from the study highlight the importance of developing, revising, and implementing consistent, coherent guidelines and policies both across and within CCGs. GPs would benefit from having accessible information of the natural history and duration of RTI that they should discuss with patients during the consultation. Suitable training for less experienced GPs could be provided with emphasis on communication and negotiation of the management of RTI at a patient and practice level.

More feedback for practices may reinforce the use of the delayed strategy. Improved national data on the prevalence of this technique could generate more interest in the approach and start to normalise the technique. The implementation of improved surveillance and tracking of prescribing is of paramount importance to reduce the number of antibiotic prescriptions in primary care. GPs may want to consider experimenting with different approaches to delayed prescribing to ascertain how best to obtain feedback on how often prescriptions are collected.

Further research could be conducted to examine the views and understanding of delayed prescribing strategies with other prescribing practitioners. Given that GPs in a primary care setting explained that their patients may access out-of-hour care services when showing RTI symptoms, it would be beneficial to know what prescribing strategies are used by out-of-hours practitioners, as well as other prescribing professionals. Moreover, investigating how GPs communicate with their patients about RTI and AMR and the influence different communication approaches have on prescribing outcomes in a consultation would be useful.

## Conclusion

While delayed antibiotic prescribing strategies are recommended in primary care when doctors feel it is safe not to prescribe immediately,^11^ it is not always successfully implemented in practice, and the use of short delays in contrast both to the evidence of natural history and the strategies used in trials. This study identifies a need for greater clarity over which method is best to delay a prescription during a consultation, and how best to operationalise delayed prescribing to create uniformity within teams and localities. Providing feedback on the uptake of antibiotics at individual and practice level may facilitate this, as well as responding to patient concern appropriately without misinterpreting the patients' agenda.

Finally, the way in which GPs communicate with patients to provide greater clarity on delayed prescribing may be beneficial. While a good rapport develops through experience, it is important to acknowledge the severity of the illness while addressing the needs of the patient. This study suggests that GPs may need guidance in how to answer the concerns of patients without interpreting these questions as a demand for antibiotics.
