# Supplementary material for: Understanding the delayed prescribing of antibiotics for respiratory tract infection in primary care: a qualitative analysis
Source: BMJ Open. 2016 Nov 18;6(11):e011882. doi: 10.1136/bmjopen-2016-011882 (PMC5129131; doi:10.1136/bmjopen-2016-011882)
Supplement: Supplementary file [file bmjopen-2016-011882supp.pdf]

Supplement 1: Interview Topic Guide

SECTION 1

a. Please describe your understanding of antibiotic prescribing for RTIs?

b. What key factors have influenced you to practice in this way?

c. How has your practice changed over time?

d. Can you please describe about your practice's policy about antibiotic prescribing and RTI?

e. How do you decide when a patient requires (or doesn't require) antibiotics for RTIs?

f. Can you tell me about the side effects of prescribing antibiotics for RTI?

SECTION 2

a. Please describe your understanding of "delayed antibiotic prescribing" as an approach to treating RTIs?

b. Why do you think delayed antibiotic prescribing is used as a way to treat RTIs?

SECTION 3

a. Please describe whether you use/don't use any form of delayed antibiotic prescribing in your practice?

b. What information do you tend to give to patients when they come to you with an RTI?

c. If you are delaying the prescription, under what circumstances do you advise patients to collect it?

d. How do patients respond to delayed antibiotic prescribing?

e. How do you reassure patients if you are delaying a prescription?

f. Please describe whether you provide patients with any information or advice for alternative ways of managing their RTI symptoms?

g. If you don't use delayed prescribing, could you tell me why this is?

h. How do other colleagues in the practice respond to delayed antibiotic prescribing?

SECTION 4

a. Please describe any facilitators of delayed antibiotic prescribing?

b. Please describe any barriers of delayed antibiotic prescribing?

SECTION 5

a. Do you think training should be given to GPs to assist them in using delayed antibiotic prescribing?

b. What type of training should be given to GPs?

c. How do you think this training should be delivered?

d. Who do you think should deliver this training?

SECTION 6

a. Is there anything else about delayed antibiotic prescribing that we haven't discussed that you think would be useful for me to know about?

NB – additional prompts were used when needed.
